# Histone demethylase JMJD3 downregulation protects against aberrant force-induced osteoarthritis through epigenetic control of NR4A1

**DOI:** 10.1038/s41368-022-00190-4

**Published:** 2022-07-14

**Authors:** Yu Jin, Zhen Liu, Zhenxia Li, Hairui Li, Cheng Zhu, Ruomei Li, Ting Zhou, Bing Fang

**Affiliations:** Department of Orthodontics, Shanghai Ninth People’s Hospital, Shanghai Jiao Tong University School of Medicine; College of Stomatology, Shanghai Jiao Tong University; National Center for Stomatology; National Clinical Research Center for Oral Diseases, Shanghai Key Laboratory of Stomatology, Shanghai, China

**Keywords:** Apoptosis, Mechanisms of disease

## Abstract

Osteoarthritis (OA) is a prevalent joint disease with no effective treatment strategies. Aberrant mechanical stimuli was demonstrated to be an essential factor for OA pathogenesis. Although multiple studies have detected potential regulatory mechanisms underlying OA and have concentrated on developing novel treatment strategies, the epigenetic control of OA remains unclear. Histone demethylase JMJD3 has been reported to mediate multiple physiological and pathological processes, including cell differentiation, proliferation, autophagy, and apoptosis. However, the regulation of JMJD3 in aberrant force-related OA and its mediatory effect on disease progression are still unknown. In this work, we confirmed the upregulation of JMJD3 in aberrant force-induced cartilage injury in vitro and in vivo. Functionally, inhibition of JMJD3 by its inhibitor, GSK-J4, or downregulation of JMJD3 by adenovirus infection of sh-JMJD3 could alleviate the aberrant force-induced chondrocyte injury. Mechanistic investigation illustrated that aberrant force induces JMJD3 expression and then demethylates H3K27me3 at the NR4A1 promoter to promote its expression. Further experiments indicated that NR4A1 can regulate chondrocyte apoptosis, cartilage degeneration, extracellular matrix degradation, and inflammatory responses. In vivo, anterior cruciate ligament transection (ACLT) was performed to construct an OA model, and the therapeutic effect of GSK-J4 was validated. More importantly, we adopted a peptide-siRNA nanoplatform to deliver si-JMJD3 into articular cartilage, and the severity of joint degeneration was remarkably mitigated. Taken together, our findings demonstrated that JMJD3 is flow-responsive and epigenetically regulates OA progression. Our work provides evidences for JMJD3 inhibition as an innovative epigenetic therapy approach for joint diseases by utilizing p5RHH-siRNA nanocomplexes.

## Introduction

Osteoarthritis (OA) is a painful degenerative joint disease that troubles millions of human beings and causes a great financial burden worldwide.^1^ To date, no effective therapeutic strategies have been developed and current treatment options are merely limited to symptom relief or late-stage surgical intervention.^2^ As a site whose main function is to carry weight and is closely related to physical activities, articular cartilage is frequently subjected to various external forces, such as stresses, strains, and pressure.^3^ However, under some circumstances, aberrant mechanical stimulation is injuries to cartilage tissues, leading to cell apoptosis, extracellular matrix degradation and cartilage degeneration.^4–7^ Specifically, joint damage, such as anterior cruciate ligament rupture or loss of meniscus integrity, could affect joint stress distribution, consequently resulting in OA initiation and progression.^8^ Therefore, aberrant mechanical force is a crucial factor for OA pathogenesis^9^ and investigation of the potential regulatory mechanism is greatly needed. Recently, an increasing number of studies have revealed the importance of epigenetics in regulating the formation and maintenance of joints.^10^

Epigenetics is the regulatory mechanism by which gene expression is altered without changing the primary DNA sequences.^11^ Specifically, there are three major epigenetic regulation types including DNA methylation, histone modifications, and non­coding regulatory RNAs.^12^ Histone modifications, including methylation, acetylation and ubiquitination, are reported to be essential for gene expression regulation and have been demonstrated to mediate various diseases through epigenetic control.^13^ Histone 3 lysine 27 (H3K27) methylation is one of the most important epigenetic events whose methylation or demethylation state participates in various biological processes, such as stem cell renewal capacity,^14^ inflammatory responses,^15^ and cancer progression.^16^ Jumonji domain-containing protein D3 (JMJD3) belongs to the JmjC histone demethylase family and regulates gene expression by demethylating H3K27me3.^17^ Multiple studies have emphasized the critical roles of JMJD3 in various human diseases, such as infectious diseases,^18^ immune diseases,^19^ developmental diseases^20^ and cancer.^21^ Moreover, JMJD3 is sensitive to external stimuli, including inflammatory cytokines, oxidative stress inducers, and carcinogenetic factors.^22,23^ Therefore, JMJD3 is a crucial demethylase involved in physiological and pathological processes.

Recently, an increasing number of studies have proposed epigenetics-based therapy methods for disease treatment, especially for tumor therapy. For example, one study demonstrated that cancer cells treated with EZH2 inhibitors are more sensitive to genotoxic stress, providing a mechanistic basis for epigenetic regulator-combined cancer therapies.^24^ Another study identified HDAC2 as a major mediator in the cancer metastatic cascade and the regulation of HDAC2 expression could be used for effective epigenetic therapies.^25^ Epigenetics-based therapeutic strategies have also been exploited in some other frontiers such as Streptococcus pneumoniae^26^ and rheumatoid arthritis.^27^ Although increasing evidence has verified the effectiveness of epigenetic therapy, studies relating epigenetic modifications with mechanical-related OA and potential therapeutic methods still remain scarce. Therefore, this study determined to investigate the epigenetic program responsible for aberrant mechanical force-induced cartilage injury and intended to explore epigenetics-based therapy approach for OA.

In the current study, we demonstrated that JMJD3 was induced in aberrant force-related cartilage injury in vitro and in vivo. Regulation of JMJD3 mediated the destructive effect of abnormal mechanical stress on chondrocytes, and further investigation suggested that the functional roles of JMJD3 may be achieved by demethylating H3K27me3 at the NR4A1 promoter. In vivo anterior cruciate ligament transection (ACLT) model validated the efficacy of GSK-J4 or si-JMJD3 administration in preventing disease progression. A peptide-siRNA nanoplatform was utilized to efficiently deliver si-JMJD3 into articular cartilage to rescue OA pathogenesis. Overall, our findings revealed an innovative epigenetic regulatory mechanism in mechanical force-related OA and implied the potential of targeting JMJD3 for OA therapy.

## Results

### Aberrant force induces JMJD3 in vitro and in vivo

**Fig. 1:**
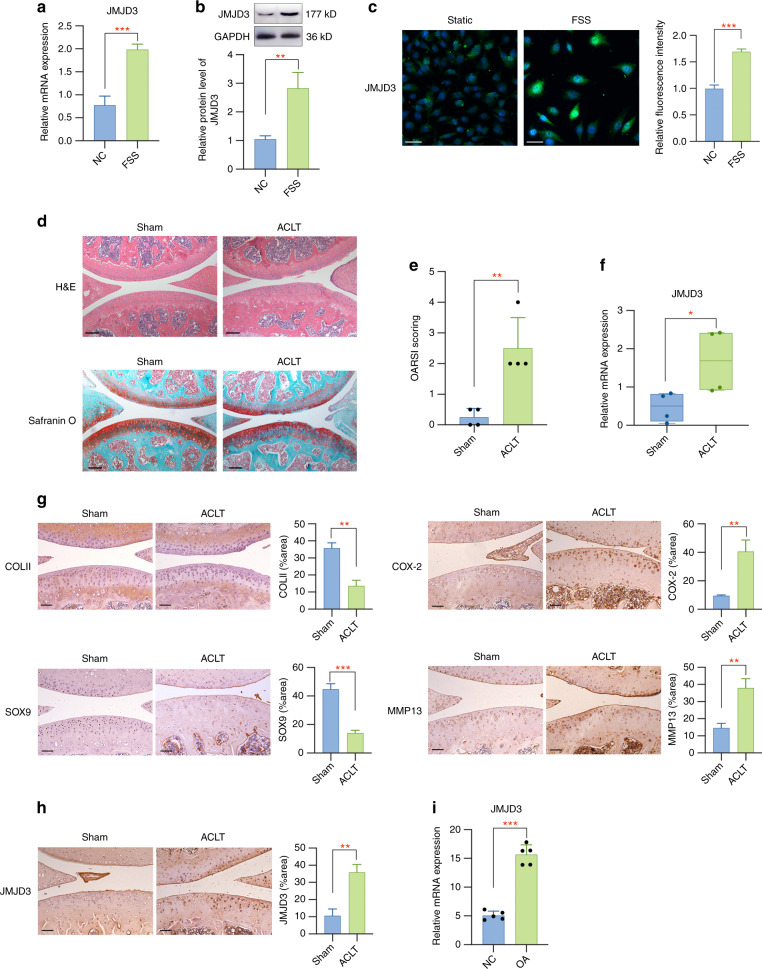
Aberrant mechanical force induces JMJD3 in vitro and in vivo. **a** Primary chondrocytes were exposed to FSS and the JMJD3 mRNA expression was evaluated by qPCR assay. **b** JMJD3 protein level was markedly increased after FSS stimulation. **c** Immunofluorescent assay presented an increased staining intensity of JMJD3 in FSS-treated chondrocytes. Scale bar: 50 μm **d** Histological analysis of joint samples from different groups. Scale bar: 100 μm **e** The OARSI scores of sham and ACLT group. **f** The mRNA level of JMJD3 in tissue samples from ACLT group was significantly upregulated when compared to that from sham controls. **g** Immunohistochemistry staining of representative markers for cartilage degradation in tissue samples from different groups. Scale bar: 50 μm **h** JMJD3 was strongly stained in ACLT mice samples. **i** A significant upregulation of JMJD3 was detected in OA tissues when compared to normal controls from GEO database. Scale bar: 50 μm *P < 0.05; **P < 0.01; ***P < 0.001

To detect whether JMJD3 participates in aberrant force-related OA, we first investigated its expression in aberrant fluid shear stress (FSS)-treated chondrocytes and OA mouse joint tissues. A significant higher mRNA level of JMJD3 was observed in FSS-stimulated chondrocytes (Fig. 1a). The protein level of JMJD3 was also upregulated in chondrocytes treated with FSS, as shown by both western blot and immunofluorescence assay (Fig. 1b, c). We established ACLT mice modal for in vivo investigation. H&E staining and safranin O fast green staining were performed for histological analysis and the results indicated that OA model was successfully constructed (Fig. 1d, e). qPCR experiments showed JMJD3 was induced in ACLT group when compared to control group (Fig. 1f). Immunohistochemistry assay results showed that the relative staining intensity of COLII and SOX9 was lower, while that of COX-2 and MMP13 was higher in OA tissues when compared to control tissues (Fig. 1g). More importantly, a strong activation of JMJD3 was manifested in OA group (Fig. 1h). Overall, the above results indicated that JMJD3 is force-sensitive and may play regulatory roles during OA pathogenesis. Furthermore, we utilized GEO public database^28^ to analyze the expression level of JMJD3 in clinical OA samples. Statistical analysis demonstrated a significant upregulation of JMJD3 in OA tissues when compared to normal controls, which supports its further functional roles in OA pathogenesis (Fig. 1i).

### Pharmacological inhibition of JMJD3 by GSK-J4 attenuates the impact of FSS on primary chondrocytes

**Fig. 2:**
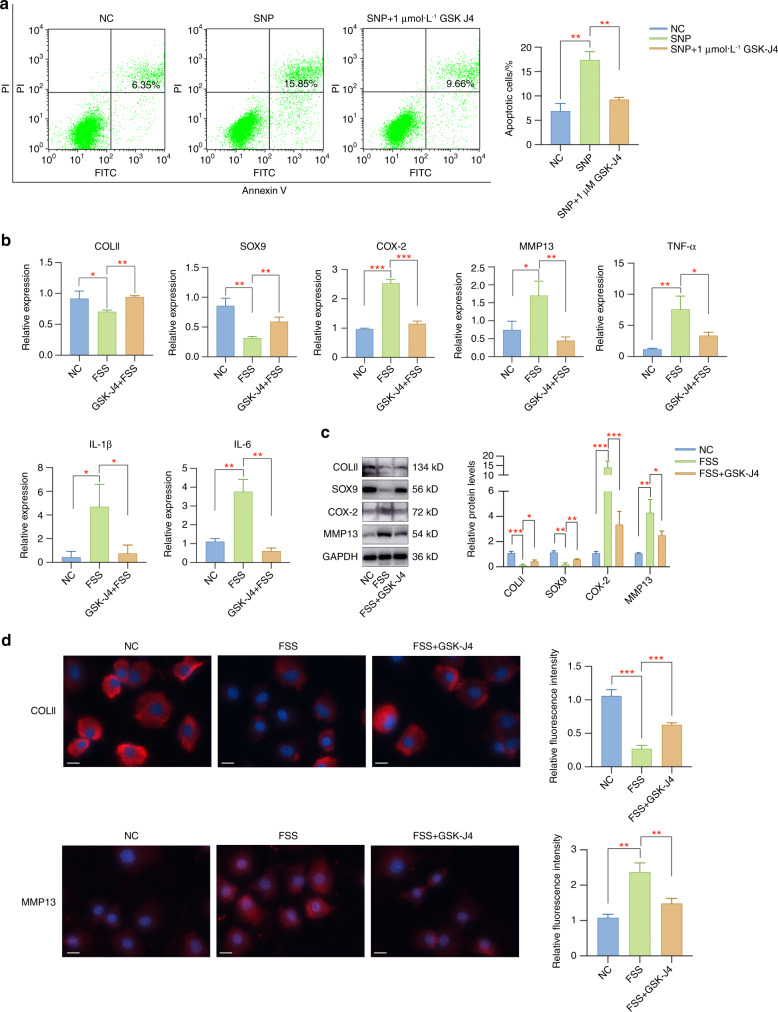
Pharmacological inhibition of JMJD3 by GSK-J4 alleviated the impact of SNP or FSS on primary chondrocytes. **a** GSK-J4 administration could rescue SNP-induced apoptosis activities in primary chondrocytes. **b** GSK-J4 treatment rescued cartilage degeneration and inflammatory responses caused by FSS. **c** The expression changes of COLII, SOX9, MMP13 and COX-2 were rescued by GSK-J4 treatment. **d** Immunofluorescent staining of COLII and MMP13 in chondrocytes with different treatments. Scale bar: 20 μm *P < 0.05; **P < 0.01; ***P < 0.001

Since we confirmed the regulation of JMJD3 by FSS treatment, we further determined to detect whether JMJD3 was involved in the responses of chondrocytes to external stimuli. Before using GSK-J4 for in vitro experiments, we performed CCK8 assay to evaluate its potential toxicity on primary chondrocytes. The results suggested that the concentration of GSK-J4 ranging from 2 to 10 μmol·L^−1^ has negative impact on chondrocyte proliferation (Fig. S1). Therefore, we chose the concentration of 1 μmol·L^−1^ GSK-J4, which could not affect cell viability, to detect its potential regulatory effect on aberrant force-stimulated chondrocytes. GSK-J4 is an inhibitor functions on the catalytic JMJ domain of KDM6B to suppress enzyme activity.^17,29^ To validate its effectiveness in inhibiting JMJD3 enzymatic activity, we treated primary chondrocytes with 1 μmol·L^−1^ GSK-J4 for 24 h and found that the expression level of H3K27me3 was significantly upregulated (Fig. S2). Based on these results, we selected 1 μmol·L^−1^ GSK-J4 for all in vitro experiments in this study. Firstly, primary chondrocytes were pretreated with or without GSK-J4 and then stimulated with sodium nitroprusside (SNP), a common reagent used to stimulate chondrocyte apoptosis. Flow cytometry results showed that the apoptosis activities were greatly induced by SNP treatment, while pretreatment with GSK-J4 effectively alleviated chondrocyte apoptosis (Fig. 2a). In addition, pharmacological inhibition of JMJD3 by GSK-J4 dramatically inhibited FSS-induced inflammatory responses, extracellular matrix degradation and cartilage degeneration (Fig. 2b–d).

### JMJD3 downregulation alleviates FSS-induced chondrocyte injury

**Fig. 3:**
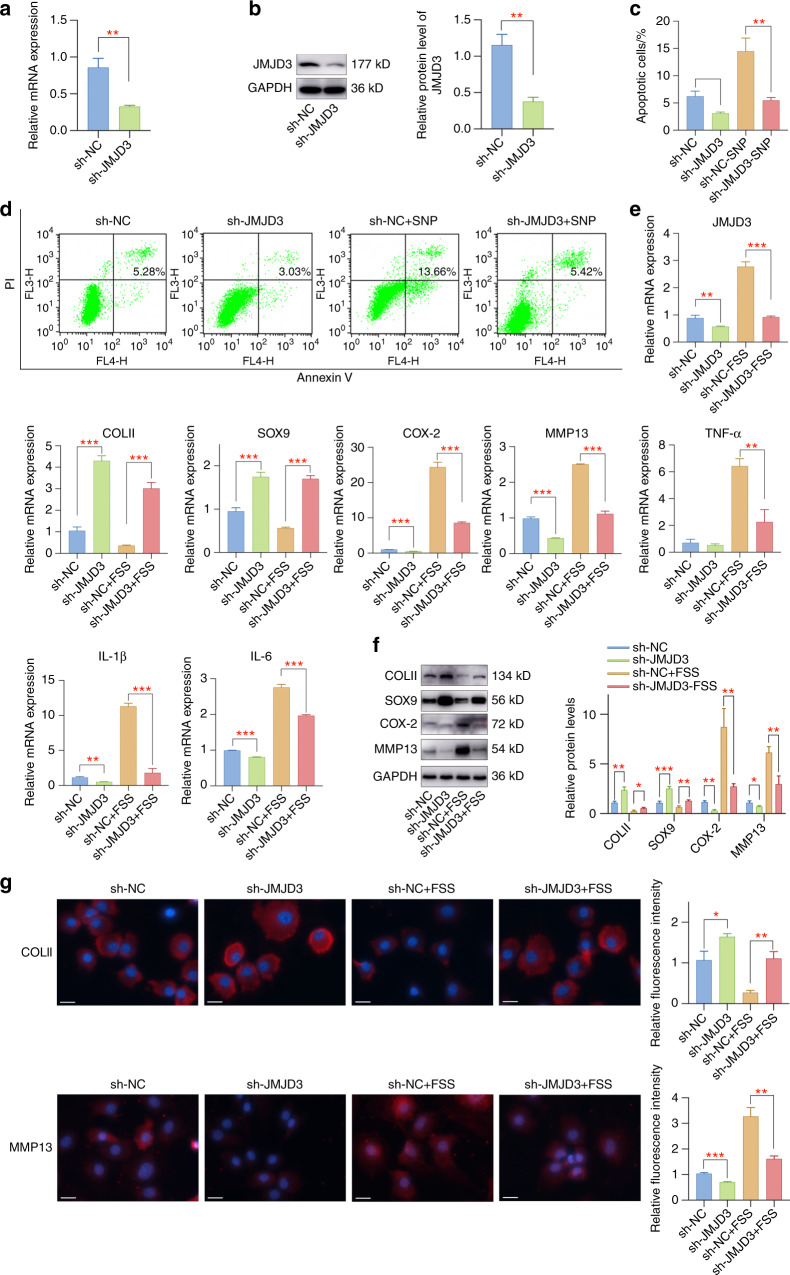
JMJD3 downregulation by adenovirus infection could rescue SNP-induced apoptosis and FSS-induced chondrocyte injury. **a** The mRNA level of JMJD3 was successfully downregulated by adenovirus infection with sh-JMJD3. **b** Downregulation of JMJD3 protein expression by adenovirus infection of sh-JMJD3. **c**, **d** JMJD3 downregulation inhibited chondrocyte apoptosis and SNP-induced apoptotic activities. **e** JMJD3 downregulation alleviated FSS-induced cartilage degeneration, extracellular matrix degradation, and inflammatory responses. **f** JMJD3 downregulation attenuated FSS-induced cartilage degeneration and extracellular matrix degradation. **g** Immunofluorescent staining of COLII and MMP13 in chondrocytes with different treatments. Scale bar: 20 μm *P < 0.05; **P < 0.01; ***P < 0.001

We utilized adenovirus infection to downregulate JMJD3 in primary chondrocytes, which was confirmed by qPCR and western blot assay (Fig. 3a, b). Further experiments suggested that JMJD3 downregulation could mediate chondrocyte apoptosis both under normal conditions and under SNP stimulation conditions (Fig. 3c, d). Specifically, JMJD3 downregulation decreased chondrocyte apoptosis and prevented SNP-induced apoptosis activities. In addition, JMJD3 downregulation improved the expression of cartilage-related markers in primary chondrocytes and rescued the destructive functions of aberrant FSS (Fig. 3e–g). These results elucidated the importance of JMJD3 in aberrant force-related OA, implying that it is a promising therapeutic target.

### Identification of NR4A1 as JMJD3-H3K27me3-target gene mediated by FSS

**Fig. 4:**
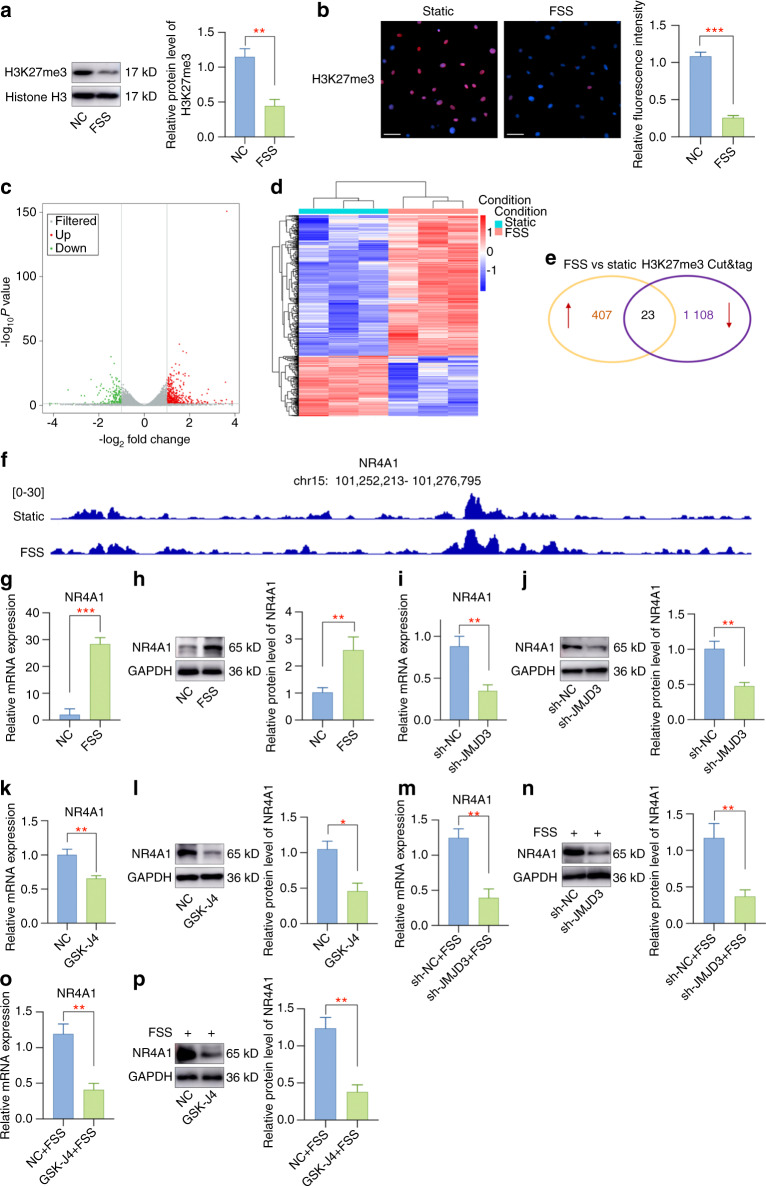
JMJD3 demethylates H3K27me3 in the promoter regions of NR4A1 during aberrant force-related cartilage injury. **a** A silence of H3K27me3 expression was presented in aberrant force-treated chondrocytes. **b** Staining intensity of H3K27me3 was decreased in chondrocytes upon FSS stimulation. **c** The volcano plot of RNA-seq was plotted to present differentially expressed genes. **d** The expression profiles of genes in chondrocytes with or without mechanical stress treatment. **e** Venn diagram analysis of RNA-seq and CUT&Tag-seq. **f** A comprehensive view of CUT&Tag sequence signals of H3K27me3 in the promoter regions of NR4A1. **g** The mRNA level of NR4A1 was greatly upregulated with FSS. **h** The protein expression of NR4A1 was elevated in chondrocytes exposed to FSS. **i** JMJD3 downregulation by adenovirus infection led to a decreased NR4A1 mRNA level. **j** JMJD3 downregulation suppressed the protein expression of NR4A1. **k** GSK-J4 administration decreased the mRNA level of NR4A1. **l** GSK-J4 administration inhibited the protein expression of NR4A1. **m** JMJD3 downregulation by adenovirus infection downregulated NR4A1 mRNA level induced by FSS treatment. **n** JMJD3 downregulation suppressed the protein expression of NR4A1 in FSS-treated chondrocytes. **o** GSK-J4 administration decreased the mRNA level of NR4A1 induced by FSS treatment. **p** GSK-J4 administration inhibited the protein expression of NR4A1 in FSS-treated chondrocytes. Scale bar: 50 μm *P < 0.05; **P < 0.01; ***P < 0.001

In most cases, JMJD3 regulates gene expression by demethylating H3K27me3 at target gene promotors.^30^ Next, we wondered whether JMJD3 functions through epigenetic control of critical genes through H3K27me3. Western blot results indicated FSS leads to a silence of H3K27me3 (Fig. 4a) and immunofluorescence staining also presented a lower intensity of H3K27me3 in FSS-treated chondrocytes (Fig. 4b). To specify the detailed JMJD3-H3K27me3-axis under FSS treatment, we combined the analysis of the transcriptomic changes in chondrocytes under FSS with H3K27me3 CUT&Tag sequencing. The volcano plot of RNA-seq was plotted to present differentially expressed genes (DEGs) (Fig. 4c). The expression profiles of genes in chondrocytes with or without FSS treatment are presented in Fig. 4d. CUT&Tag sequencing analysis results showed that 1131 genes may be directly mediated by H3K27me3. A combined analysis of RNA-seq and CUT&Tag-seq identified 23 genes that may act as potential target genes (Fig. 4e). We then chose to focus on NR4A1, whose fold-change was the most evident. The peaks of NR4A1 levels in samples from different groups are displayed in Fig. 4f, and JMJD3 upregulation induced by FSS decreased the H3K27me3 level at the promoter region of NR4A1. Subsequently, further experiments verified that NR4A1 is a flow-responsive gene that can be upregulated by high shear stress (Fig. 4g, h). To further investigate whether the regulation of NR4A1 by H3K27me3 silencing was mediated by JMJD3, NR4A1 expression was detected in cells infected with sh-NC or sh-JMJD3. The remarkably lower expression of NR4A1 confirmed the regulatory roles of JMJD3 on NR4A1 (Fig. 4i, j). Moreover, GSK-J4 treatment also suppressed the expression of NR4A1 in primary chondrocytes (Fig. 4k, l). Subsequently, to further analyze whether JMJD3 gene silencing affects the regulatory effect of FSS on NR4A1, we performed in vitro rescue experiments. Specifically, primary chondrocytes infected with sh-NC/sh-JMJD3 or pretreated with or without GSK-J4 were subjected to FSS treatment. After 2 h of FSS treatment, total RNA and protein were extracted, and qPCR and western blot assay were performed to detect the expression level of NR4A1. The results indicated that JMJD3 silencing could effectively alleviate the induction of NR4A1 by FSS stimulation (Fig. 4m–p). Therefore, these findings support our conclusion that JMJD3 mediates aberrant force-induced OA through epigenetic control of NR4A1.

### NR4A1 regulates the biological phenotypes of primary chondrocytes and FSS-induced OA-like pathological changes

**Fig. 5:**
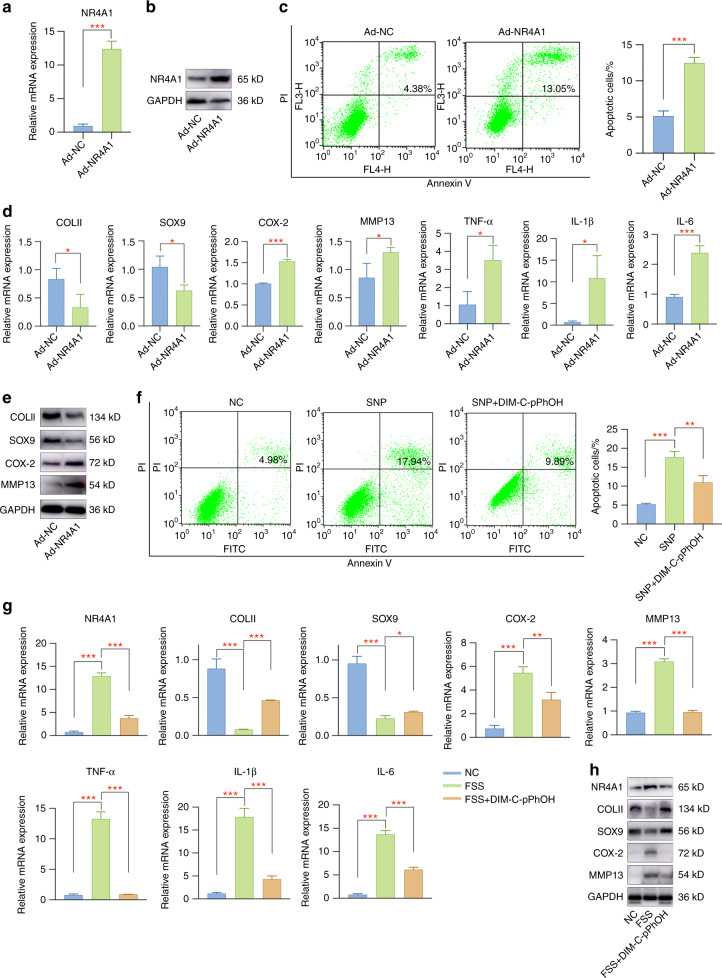
NR4A1 regulates the physiological activities of primary chondrocytes and FSS-induced OA-like pathological changes. **a** The mRNA level of NR4A1 was significantly upregulated by adenovirus infection. **b** The protein level of NR4A1 was remarkably elevated in Ad-NR4A1-infected chondrocytes. **c** Overexpression of NR4A1 induced chondrocyte apoptosis. **d** NR4A1 overexpression caused cartilage degeneration, extracellular matrix degradation and inflammatory mediators production. **e** NR4A1 overexpression induced cartilage pathogenesis. **f** NR4A1 inhibition by DIM-C-pPhOH treatment partially suppressed SNP-induced apoptosis. **g** Pretreatment with DIM-C-pPhOH alleviated FSS-induced cartilage injury. **h** Cartilage pathogenesis caused by aberrant force stimulation was rescued by DIM-C-pPhOH. *P < 0.05; **P < 0.01; ***P < 0.001

Firstly, the biological effect of NR4A1 on chondrocytes was investigated by Ad-NR4A1 infection. NR4A1 was successfully overexpressed in Ad-NR4A1-infected chondrocytes when compared to the Ad-NC group (Fig. 5a, b, Fig. S3). Apoptosis assay demonstrated that NR4A1 induced apoptosis activities in chondrocytes (Fig. 5c). Furthermore, NR4A1 overexpression inhibited COLII and SOX9 while promoted COX-2 and MMP13 expression (Fig. 5d, e, Fig. S4). Proinflammatory mediators (TNF-α, IL-1β, and IL-6) were greatly induced in Ad-NR4A1 group (Fig. 5d). Afterwards, we intended to detect whether NR4A1 was involved in FSS-related OA pathogenesis. Chondrocytes were pretreated with the NR4A1 inhibitor DIM-C-pPhOH and then subjected to FSS treatment. The results showed that NR4A1 inhibition by DIM-C-pPhOH effectively alleviated SNP-induced apoptosis (Fig. 5f) and partially rescued OA-like pathological changes (Fig. 5g, h, Fig. S5).

### NR4A1 mediates chondrocytes through targeting Akt pathway

**Fig. 6:**
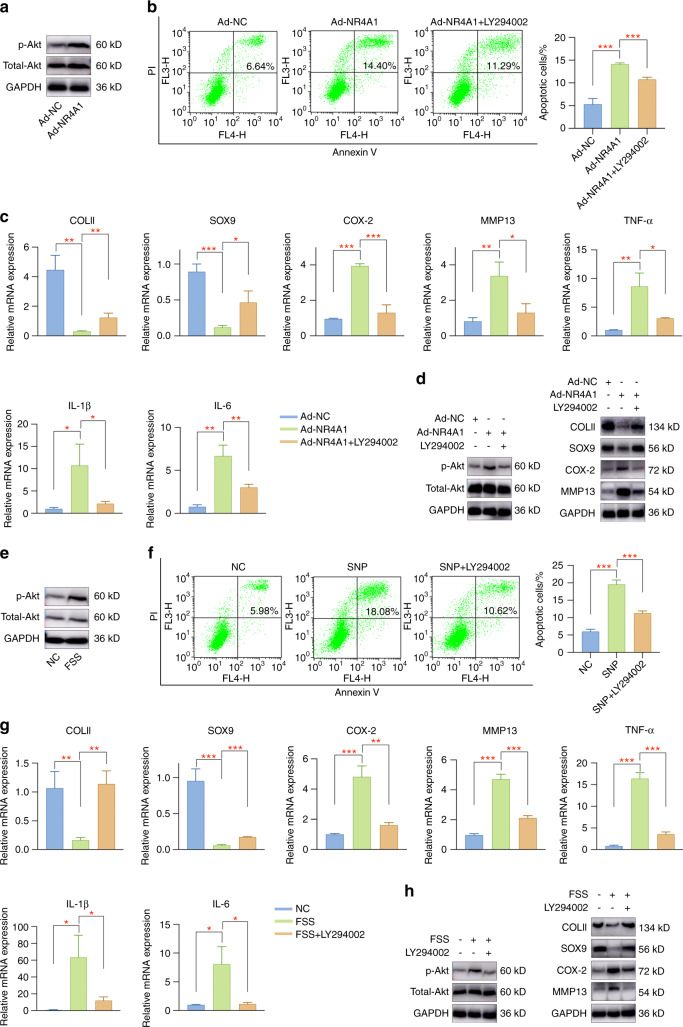
Akt signaling participates in aberrant force-mediated OA pathogenesis. **a** Akt signaling was activated in Ad-NR4A1-infected chondrocytes. **b** Treatment with Akt pathway inhibitor LY294002 effectively inhibited chondrocyte apoptosis induced by NR4A1. **c** Akt pathway inhibition partially rescued NR4A1-induced chondrocyte injury. **d** Representative markers for OA evaluation were investigated by western blot assay. **e** Aberrant mechanical force induced Akt signaling pathway activation. **f** Pretreatment with LY294002 prevented SNP-induced apoptosis activities in primary chondrocytes. **g** Akt pathway inhibition by LY294002 partially attenuated FSS-induced chondrocyte injury. **h** Representative markers for evaluating cartilage pathogenesis were detected. *P < 0.05; **P < 0.01; ***P < 0.001

Published studies have reported that NF-κB and Akt pathways participate in articular cartilage pathogenesis,^31–33^ and NR4A1 was demonstrated to mediate NF-κB or Akt signaling in different biological processes.^34–36^ Therefore, we investigated whether NR4A1 functions through targeting NF-κB or Akt pathways in primary chondrocytes. Representative marker proteins in the selected pathways were evaluated by western blot assay. An obvious activation of Akt signaling was observed in NR4A1-overexpressing chondrocytes, while no evident changes occurred in NF-κB pathway (Fig. 6a, Fig. S6, S7). Then, the specific Akt pathway inhibitor LY294002 was utilized to confirm that NR4A1 functions through targeting Akt signaling. As shown in Fig. 6b–d and Fig. S8, LY294002 pretreatment rescued NR4A1-induced chondrocyte injury, mainly by suppressing cell apoptosis, improving cartilage differentiation-related marker levels, decreasing extracellular matrix degradation, and alleviating inflammatory responses. The above findings illustrated that the regulatory functions of NR4A1 were achieved through targeting Akt pathway.

### Akt signaling pathway participates in FSS-induced OA pathogenesis

To detect the regulatory roles of Akt pathway in FSS-induced OA, we examined marker proteins in Akt signaling. Western blot analysis indicated Akt pathway activation in FSS-treated cells, as demonstrated by an elevated level of p-Akt (Fig. 6e, Fig. S9). Subsequently, the specific Akt pathway inhibitor LY294002 was used for rescue experiments. The results indicated that LY294002 administration significantly reduced SNP-induced apoptosis activities (Fig. 6f) and alleviated aberrant force-induced chondrocyte degeneration (Fig. 6g, h, Fig. S10). Therefore, we speculated that JMJD3 regulates aberrant force-related OA pathogenesis through H3K27me3-NR4A1-mediated Akt signaling activation.

### Epigenetic control of JMJD3 effectively alleviates OA pathogenesis

**Fig. 7:**
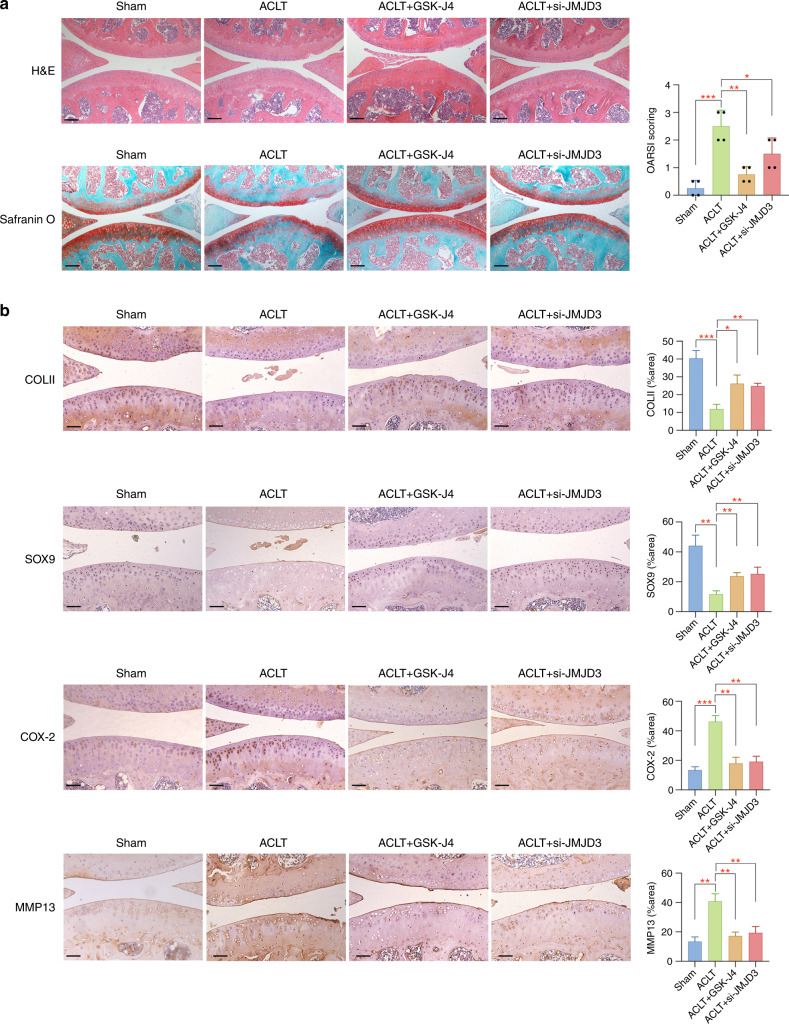
Administration of GSK-J4 or si-JMJD3 nanocomplex effectively alleviates OA pathogenesis. **a** Histological analysis of tissue samples and corresponding OARSI scores in different groups. Scale bar: 100 μm (**b**) Immunohistochemistry staining of COLII, SOX9, COX-2, and MMP13 for evaluating cartilage pathogenesis in tissue samples from mice with different treatments. Scale bar: 50 μm *P < 0.05; **P < 0.01; ***P < 0.001

**Fig. 8:**
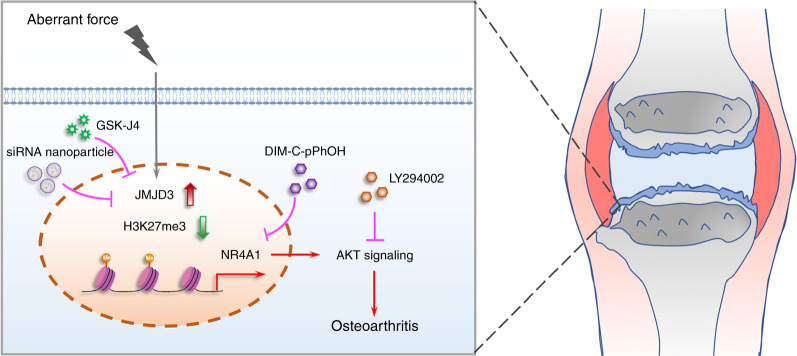
A schematic view illustrating the epigenetic regulatory mechanism of JMJD3 in aberrant mechanical force-related OA

To confirm the in vivo effect of JMJD3 regulation on mechanical-related OA pathogenesis, we constructed ACLT OA mice model and randomly allocated mice to the following four groups: Group I (Sham, *n* = 4) mice did not undergo any treatment. Group II (ACLT, *n* = 4) mice underwent ACLT surgery. Group III (ACLT + GSK-J4, *n* = 4) mice underwent ACLT surgery and injection with GSK-J4 (10 mg·kg^−1^, 10 μL, twice per week) in the articular regions. Group IV (ACLT + si-JMJD3, *n* = 4) mice underwent ACLT surgery and injection with JMJD3 siRNA nanocomplex (500 nmol·L^−1^, 10 μL, twice per week) in the articular regions. Samples were collected at 8 weeks after surgery. Histological analysis demonstrated obvious cartilage degradation in OA group while GSK-J4 or si-JMJD3 nanocomplex administration partially rescued this effect (Fig. 7a). More specifically, safranin O-positive cells were markedly decreased in OA model, suggesting that ACLT-induced OA resulted in a loss of glycosaminoglycan. Mice with ACLT surgery after GSK-J4 or si-JMJD3 nanocomplex administration presented less severe cartilage injury (Fig. 7a). For immunohistochemistry assay, the changes of cartilage-related markers and extracellular matrix degradation proteins were reversed by the injection of GSK-J4 or si-JMJD3 nanocomplex (Fig. 7b). The overall epigenetic-based regulatory mechanisms of JMJD3 in alleviating mechanical stress-related OA are presented in Fig. 8.

## Discussion

OA is a prevalent disease that seriously troubles human beings, leading to impaired life quality and great physical and psychological burdens.^37^ However, due to the complex pathological factors and pathogenesis process, there still remain no effective approaches for OA treatment. As a joint featured by high load-bearing properties and closely related to daily activities, cells in the articular tissues are typically subjected to various mechanical stimuli.^3^ Therefore, the investigation of how cells within the articular tissues sense or respond to external mechanical forces is an area of intense research. In contrast to other studies that used proinflammatory mediators, such as IL1β, to induce OA, we exerted abnormal mechanical stress on chondrocytes in vitro to simulate more physiological-like conditions of the OA environment and generated ACLT mice model to mimic OA initiation and progression in vivo. We aimed to elucidate novel mechanisms in aberrant force-induced OA to provide evidences for innovative therapeutic methods.

Till now, epigenetic events have been reported to occur in many processes, including tumorigenesis,^38^ stem cell survival and differentiation,^39^ immunity,^40^ and other diseases,^41^ while comprehensive investigation of epigenetic events underlying mechanical stress-induced OA pathogenesis is still lacking. Several studies have uncovered the involvement of epigenetic regulation in mechanical force-related biological activities or diseases. For example, a study found that histone methyltransferase EZH2 was suppressed in response to FSS and endothelial cells entered into a state of cell quiescence.^42^ Another study demonstrated a mechanical force-sensitive lncRNA SNHG8, which could inhibit osteogenic differentiation by regulating EZH2 in periodontal ligament stem cells.^43^ Moreover, a study illustrated the associations between mechanical forces and skeletal epigenetic changes, which resulted in remarkable alterations in bone cell transcriptional activity.^44^ Investigations regarding the epigenetics underlying mechanical force-related OA remain scarce. A study indicated that long non-coding RNA HOTAIR was greatly induced upon mechanical stimulation and it could interact with miR-221 to target BBC3. HOTAIR inhibition effectively protected chondrocytes from apoptosis induced by mechanical force, serving as a therapeutic target.^45^ In addition, it was reported that excessive mechanical stress increased the binding of BRD4 to H3K27ac on the promoter region of Trem1, leading to TMJ OA-like pathological changes.^46^ These findings provided evidences for targeting BRD4 as a promising strategy for OA therapy. In the present study, we explored the potential epigenetic regulation mechanism during aberrant force-induced OA.

Histone modifications among which H3K27 methylation is a major player in multiple biological processes, and JMJD3 is a major histone demethylase which catalyzes the removal of H3K27me3.^30^ It was demonstrated that JMJD3 could be regulated by multiple kinds of external cues and signals from different cell types. Also, it participates in a wide array of physiological and pathological processes. For example, JMJD3 was demonstrated to demethylate H3K27me3 in the promotor site of Nfatc1 gene, which is essential for osteoclast differentiation.^47^ In addition, JMJD3-induced H3K27me3 regulation acts as a crucial epigenetic modulator during CD4 T cell activation, implying its potential roles in immune responses.^48,49^ More importantly, a variety of studies have identified the functions of JMJD3 in inflammation. JMJD3 downregulation or GSK-J4 treatment suppressed inflammatory reactions in IL-1β-treated synovial fibroblasts, and this effect was obtained mainly by controlling the methylation status of H3K27me3 at the promoter of target genes.^27^ In macrophages, JMJD3 knockdown or GSK-J4 treatment significantly inhibited NLRP3 inflammasome activation and proinflammatory cytokine production caused by lipopolysaccharide stimulation.^29,50^ Moreover, GSK-J4 was also illustrated to limit inflammation by inducing a tolerogenic phenotype in dendritic cells.^51^ The above findings identified the critical role of JMJD3 as a mediator in inflammatory responses and that it is a promising epigenetic therapeutic target for disease treatment. Therefore, in our study, we discovered the ectopic expression of JMJD3 upon mechanical stimulation and, thus, wondered whether JMJD3 was involved in aberrant force-related OA. Through a series of experiments, we uncovered the functions of JMJD3 modulation in mechanical stress-induced chondrocyte injury and the effectiveness of the administration of GSK-J4 or si-JMJD3 for OA therapy. In our work, we adopted a peptide-siRNA nanoplatform which was previously published for efficient siRNA delivery for chondrocyte injury.^52–54^ This siRNA nanocomplex had the advantage of specifically targeting inflammation in the joint without off-target toxicities. si-JMJD3 was successfully loaded on the peptide-siRNA nanoplatform and penetrated into injured cartilage for epigenetic-based therapy.

Subsequently, to further elucidate the epigenetic-based regulatory mechanism of JMJD3 in OA, a combination of RNA-seq analysis and CUT&Tag-seq analysis was conducted, and the JMJD3-H3K27me3-NR4A1 axis was finally identified. NR4A1, also known as Nur77, is an important transcription factor that is closely associated with multiple cell biological phenotypes, such as cell growth and apoptosis.^55,56^ For example, a study demonstrated that inflammation induced NR4A1 expression, consequently resulting in the activation of TGF-β/SMAD-mediated breast cancer cell migration, invasion, and metastasis.^57^ In mesenchymal stem cells, elevated NR4A1 expression led to increased cytokine and growth factor production, which was closely associated with local immune response modulation.^58^ More importantly, previous findings identified that NR4A1 is characterized by immediate early genes that could be induced by diverse kinds of stimuli, including growth factors, cytokines, inflammatory and physiological stimuli.^59^ Moreover, NR4A1 was also suggested to sense changes in the cellular microenvironment to mediate physiological and pathological processes. An in vitro study showed an immediate induction of NR4A1 in macrophages or monocytes after lipopolysaccharide treatment or other inflammatory stimuli. Another study demonstrated that pharmacological activation or NR4A1 overexpression effectively suppressed the production of inflammatory cytokines and chemokines. However, some studies found that NR4A1 binds to mitochondria and promotes cell apoptosis and death.^60^ From our perspective, the opposite effects of NR4A1 on cell inflammation and survival may due to the fact that NR4A1 acts its different functions dependent on different cell types or specific circumstances. A study demonstrated an elevated level of NR4A1 in OA, and NR4A1 knockdown decreased cleaved PARP1 expression and alleviated the apoptosis of OA chondrocytes.^61^ Zheng et al. also revealed that NR4A1 significantly promotes TNF-α-induced chondrocyte death via mitochondrial fission activation.^62^ The above findings suggested that NR4A1 has a negative impact on chondrocytes, which was in consistent with our results. In our study, NR4A1 rapidly responded to FSS, and the inhibition of NR4A1 rescued aberrant force-related OA pathogenesis. Further experiments implied the regulatory functions of NR4A1 may be achieved by targeting Akt signaling. These findings revealed the JMJD3-H3K27me3-NR4A1-Akt axis underlying aberrant force-induced OA pathogenesis. More importantly, we provided evidences for the prospect of epigenetic-based therapy for OA in the future. Intraarticular injection of GSK-J4 or si-JMJD3 nanocomplex remarkably alleviated OA progression.

In summary, our study illustrated that epigenetic regulation is closely involved in mechanical-related OA diseases. JMJD3 is mechanical stress-responsive and functions through controlling the demethylation of H3K27me3 in the promoter region of NR4A1. Inhibition or downregulation of JMJD3 effectively rescued mechanical force-induced chondrocyte injury. An efficient siRNA nanoplatform was used for si-JMJD3 delivery, and the therapeutic effect was validated. Our findings provide insights into the epigenetic regulatory mechanism in mechanical-induced OA and indicate the great potential for the discovery of new epigenetics-based remedies for OA. However, there still exist some limitations in the present study. First, the in vivo experiments we performed for evaluating the effect of targeting JMJD3 on treating OA was based on small animals. Additionally, no investigation was conducted to explore the underlying mechanism of FSS-induced JMJD3 upregulation. Large animal models or clinical trials may be conducted to validate the therapeutic effect of JMJD3 downregulation on OA. More comprehensive studies will be performed to illuminate the upstream signaling of JMJD3 induction caused by FSS in the future.

## Materials and methods

### Public database

To detect the expression of JMJD3 in clinical OA samples, we downloaded GSE1919 dataset from GEO (http://www.ncbi.nlm.nih.gov/geo/) database, a public repository for high-throughput microarray and next-generation sequence datasets.^28^ We compared the expression level of JMJD3 in OA samples and normal controls.

### Primary chondrocyte isolation and culture

Primary chondrocytes were isolated from C57BL/6 J mice (3-week-old). Specifically, cartilage tissues were obtained and first digested for 30 min in 0.25% trypsin-EDTA at 37 °C. Cartilage tissues were then cut into 1 mm^3^ pieces and digested in 0.2% collagenase II for ~3 h at 37 °C. Afterwards, chondrocytes were collected and resuspended in DMEM/F12 supplemented with 10% fetal bovine serum and 1% penicillin-streptomycin (Gibco, USA). Primary chondrocytes from passage 1–3 were used for the following experiments.

### In vitro FSS experiment

Aberrant mechanical force was performed by a Flexcell FX-4000 strain unit (Flexcell, FX4000, Burlington, Ontario, Canada) as previously stated.^63^ Primary chondrocytes in this study were treated with 20 dyne per cm^2^ FSS for 2 h and collected for further analysis.

### RNA isolation and qRT-PCR

RNA was extracted by TRIzol reagent (Takara, Japan) and reverse transcription was complemented by using a PrimeScript (Takara, Japan). Quantitative reverse transcriptase-polymerase chain reaction (qRT-PCR) was performed on a Light Cycler 480 II (Roche) with SYBR Green Mix (Takara, Japan). All the primers were synthesized by Sangon Biotech (Shanghai) and the detailed sequences were listed in Table S1. The data were subjected to analysis based on the 2^−ΔΔCt^ method with GAPDH acting as the housekeeping gene.

### RNA sequencing and differential expression analysis

RNA libraries were constructed by a TruSeq Stranded mRNA LT Sample Prep Kit (Illumina, San Diego, CA, USA) as instructed. OE Biotech Co., Ltd. (Shanghai, China) conducted RNA-seq and differential expression analysis involved in this study. The sequence platform is Illumina HiSeq X Ten platform and differential expression analysis was performed through the DESeq (2012) R package. Genes that met the threshold of *P* < 0.05 and |logFC | >1 were determined as DEGs.

### Western blot assay

Chondrocytes underwent lysis with SDS lysis buffer (Beyotime, China). A routine procedure was conducted for western blotting as previously described.^63^ Primary antibodies against COLII (Proteintech, USA), SOX9 (Abcam, USA), COX-2 (CST, UK), MMP13 (Proteintech, USA), H3K27me3 (CST, UK), Histone H3 (CST, UK), NR4A1 (ABclonal, USA), JMJD3 (ABclonal, USA), p-p65 (CST, UK), p65 (CST, UK), IκBα (CST, UK), p-Akt (CST, UK), Akt (CST, UK) and GAPDH (Proteintech, USA) were incubated with PVDF membranes at 4 °C overnight. After incubating with the secondary antibody for 1 h, the signals were observed on an Amersham 600 Chemiluminescence System. The relative intensity of protein band was analyzed by ImageJ.

### Immunofluorescence assay

Samples were immobilized with 4% paraformaldehyde, permeabilized in 0.1% Triton X-100 and blocked in 3% BSA. Subsequently, the fixed cells were incubated with primary antibodies against H3K27me3 (CST, UK), JMJD3 (ABclonal, USA), COLII (Proteintech, USA) and MMP13 (Proteintech, USA) overnight at 4 °C. Samples were then incubated with Alexa Fluor-conjugated secondary antibodies at room temperature protected from light. Cells were observed and images were captured by an inverted fluorescence microscope (ZEISS, Germany).

### In vitro small molecular inhibitor treatment

GSK-J4, DIM-C-pPhOH, and LY294002 (Sellect, USA) were dissolved in DMSO at a storage concentration and stored at −80 °C until use. Primary chondrocytes were pretreated with 1 μmol·L^−1^ GSK-J4, 20 μmol·L^−1^ DIM-C-pPhOH, or 10 μmol·L^−1^ LY294002 for 24 h and then subjected to FSS or SNP treatment.

### Adenovirus knockdown of JMJD3

sh-JMJD3 adenovirus was provided by HanBio Technology (Shanghai, China). Chondrocytes were infected with sh-JMJD3 adenovirus at a multiplicity of 300 when the cell density reached ~30%–50%. The culture medium was replaced after 6–8 h upon adenovirus infection.

### p5RHH-siRNA nanocomplex

We utilized a cationic amphipathic peptide, named p5RHH peptide to deliver siRNA into articular cartilage as previously described.^52–54^ The p5RHH peptide was synthesized by Genscript (Nanjing, China) and si-JMJD3 was provided by HanBio Technology (Shanghai, China). The detailed procedures for preparing p5RHH-siRNA nanocomplex are as followed. A concentration of 10 mmol·L^−1^ p5RHH peptide and 100 μmol·L^−1^ si-JMJD3 were mixed at a ratio of peptide:si-JMJD3 as 100:1 in HBSS. The mixture was then incubated on ice for 10 min, after which it was injected into articular regions at a final siRNA concentration of 500 nmol·L^−1^.

### Flow cytometry assay

Apoptosis assay in this study was conducted by flow cytometry. Briefly, chondrocytes were pretreated with or without 1 μmol·L^−1^ GSK-J4 or 20 μmol·L^−1^ DIM-C-pPhOH after which stimulated with 500 μmol·L^−1^ SNP for 24 h. Samples were harvested and stained by an Annexin V-APC/PI Apoptosis Detection Kit (KeyGEN BioTECH, China). Stained samples were analyzed on a flow cytometer (BD Biosciences, USA).

### CUT&Tag library generation and sequencing

CUT&Tag library was generated by NovoNGS CUT&Tag 3.0 High-Sensitivity Kit (for Illumina) (Novoprotein, China) as previously stated.^63^ Specifically, collected cells were incubated with prewashed ConA beads and then probed with H3K27me3 (CST, UK) on a rotator for a more complete reaction. Secondary antibody was then added into samples for reacting about 1 h. Afterwards, samples were cultured with hyperactive pAG-Tn5 transposon at room temperature for 1 h. Tagmentation was performed under the incubation with tagmentation buffer at 37 °C, after which the reaction was stopped by adding 10% SDS and incubated at 55 °C. DNA was then extracted by using phenol-chloroform and ethanol precipitation method, and PCR was conducted to amplify the libraries. CUT&Tag-seq was finished on Illumina Novaseq (PE150) platform.

### In vivo OA animal model

The animals used in this study were approved by the Ethics Board of Shanghai Ninth People’s Hospital affiliated to Shanghai Jiao Tong University School of Medicine. In this study, OA was induced by ACLT surgery in 12-week-old C57BL/6 mice as previously indicated.^64^ A total of 16 mice were included and mice were distributed to the following four groups (*n* = 4 per group): sham-operated group, ACLT-induction, ACLT + GSK-J4 and ACLT + si-JMJD3. Specifically, ACLT was performed on the left knee joint of C57BL/6 mice and raised for 2 weeks. GSK-J4 or si-JMJD3 nanocomplex was then administered into the joint space twice per week for 6 consecutive weeks. At the 8th week after ACLT operation, mice were sacrificed and joint tissues were immediately collected.

### Histological analysis

Collected tissues were fixed in 4% paraformaldehyde and then decalcified in 10% ethylenediaminetetraacetic acid solution for 3 weeks. Demineralized samples were embedded in paraffin after gradient dehydration and cut into 5 μm thick sections. For histological analysis, samples were stained with H&E and safranin-O-fast green as the protocols indicated. The severity of OA was determined by using the Osteoarthritis Research Society International (OARSI) scoring system as the criteria.^65^

### Immunohistochemistry (IHC) assay

5 mm thick-slides were deparaffinized, hydrated and submerged into proteinase K for antigen retrieval. The slides were incubated with COLII (Proteintech, USA), SOX9 (CST, UK), COX-2 (CST, UK), MMP13 (Proteintech, USA), and JMJD3 (ABclonal, USA) at 4 °C overnight. The sections were then probed with HRP-conjugated secondary antibodies, stained with hematoxylin, dehydrated, cleared and mounted. Images were captured and the relative staining intensity was quantitatively calculated by ImageJ software.

### Statistical analysis

Statistical analysis was performed by using GraphPad Prism 8.0 software throughout this study. Analysis of two or more groups was conducted by Student’s *t* test or one-way ANOVA, and values of *P* < 0.05 were considered as statistically significant in all analyses.

## Supplementary information

supplemental materials

## References

[1] Lu KH (2021). The potential remedy of melatonin on osteoarthritis. J. Pineal Res..

[2] Li X (2021). Nanoparticle-Cartilage Interaction: Pathology-Based Intra-articular Drug Delivery for Osteoarthritis Therapy. Nanomicro Lett..

[3] Yokota H, Goldring MB, Sun HB (2003). CITED2-mediated regulation of MMP-1 and MMP-13 in human chondrocytes under flow shear. J. Biol. Chem..

[4] Buckwalter JA, Martin JA, Brown TD (2006). Perspectives on chondrocyte mechanobiology and osteoarthritis. Biorheology.

[5] Kerin A, Patwari P, Kuettner K, Cole A, Grodzinsky A (2002). Molecular basis of osteoarthritis: biomechanical aspects. Cell Mol. Life Sci..

[6] Carter DR, Wong M (2003). Modelling cartilage mechanobiology. Philos. Trans. R. Soc. Lond. B Biol. Sci..

[7] Carter DR (2004). The mechanobiology of articular cartilage development and degeneration. Clin. Orthop. Relat. Res..

[8] Donahue TL, Fisher MB, Maher SA (2015). Meniscus mechanics and mechanobiology. J. Biomech..

[9] Guilak F (2011). Biomechanical factors in osteoarthritis. Best. Pr. Res. Clin. Rheumatol..

[10] Rice SJ, Beier F, Young DA, Loughlin J (2020). Interplay between genetics and epigenetics in osteoarthritis. Nat. Rev. Rheumatol..

[11] Allis CD, Jenuwein T (2016). The molecular hallmarks of epigenetic control. Nat. Rev. Genet..

[12] Lorzadeh A, Romero-Wolf M, Goel A, Jadhav U (2021). Epigenetic Regulation of Intestinal Stem Cells and Disease: A Balancing Act of DNA and Histone Methylation. Gastroenterology.

[13] Greer EL, Shi Y (2012). Histone methylation: a dynamic mark in health, disease and inheritance. Nat. Rev. Genet..

[14] Wei, Y. et al. Efficient derivation of human trophoblast stem cells from primed pluripotent stem cells. *Sci. Adv.* **7**, 10.1126/sciadv.abf4416 (2021). 10.1126/sciadv.abf4416 PMC8357231 34380613

[15] Wu C (2020). Interplay of m(6)A and H3K27 trimethylation restrains inflammation during bacterial infection. Sci. Adv..

[16] Suwala AK (2022). Oligosarcomas, IDH-mutant are distinct and aggressive. Acta Neuropathol..

[17] Xiang Y (2007). JMJD3 is a histone H3K27 demethylase. Cell Res..

[18] Nguyen K (2021). Inhibition of the H3K27 demethylase UTX enhances the epigenetic silencing of HIV proviruses and induces HIV-1 DNA hypermethylation but fails to permanently block HIV reactivation. PLoS Pathog..

[19] Sun D, Cao X, Wang C (2019). Polycomb chromobox Cbx2 enhances antiviral innate immunity by promoting Jmjd3-mediated demethylation of H3K27 at the Ifnb promoter. Protein Cell.

[20] Ding Y (2021). JMJD3: a critical epigenetic regulator in stem cell fate. Cell Commun. Signal.

[21] Lee SH, Kim O, Kim HJ, Hwangbo C, Lee JH (2021). Epigenetic regulation of TGF-beta-induced EMT by JMJD3/KDM6B histone H3K27 demethylase. Oncogenesis.

[22] Salminen A, Kaarniranta K, Hiltunen M, Kauppinen A (2014). Histone demethylase Jumonji D3 (JMJD3/KDM6B) at the nexus of epigenetic regulation of inflammation and the aging process. J. Mol. Med (Berl.).

[23] Merkwirth C (2016). Two Conserved Histone Demethylases Regulate Mitochondrial Stress-Induced Longevity. Cell.

[24] Liao, Y. et al. Inhibition of EZH2 transactivation function sensitizes solid tumors to genotoxic stress. *Proc. Natl Acad. Sci. USA* **119**, 10.1073/pnas.2105898119 (2022). 10.1073/pnas.2105898119 PMC8784159 35031563

[25] Krauss, L. et al. HDAC2 facilitates pancreatic cancer metastasis. *Cancer Res*. 10.1158/0008-5472.CAN-20-3209 (2021).

[26] Connor MG (2021). The histone demethylase KDM6B fine-tunes the host response to Streptococcus pneumoniae. Nat. Microbiol..

[27] Wu W (2019). Cystathionine-gamma-lyase ameliorates the histone demethylase JMJD3-mediated autoimmune response in rheumatoid arthritis. Cell Mol. Immunol..

[28] Barrett T (2013). NCBI GEO: archive for functional genomics data sets-update. Nucleic Acids Res..

[29] Kruidenier L (2012). A selective jumonji H3K27 demethylase inhibitor modulates the proinflammatory macrophage response. Nature.

[30] Zhang X, Liu L, Yuan X, Wei Y, Wei X (2019). JMJD3 in the regulation of human diseases. Protein Cell.

[31] Saito T (2010). Transcriptional regulation of endochondral ossification by HIF-2alpha during skeletal growth and osteoarthritis development. Nat. Med..

[32] Yang S (2010). Hypoxia-inducible factor-2alpha is a catabolic regulator of osteoarthritic cartilage destruction. Nat. Med..

[33] Li ZC (2014). Functional annotation of rheumatoid arthritis and osteoarthritis associated genes by integrative genome-wide gene expression profiling analysis. PLoS ONE.

[34] Wang S (2021). HUCMSCs transplantation combined with ultrashort wave therapy attenuates neuroinflammation in spinal cord injury through NUR77/ NF-kappaB pathway. Life Sci..

[35] Cao J (2021). NR4A1 knockdown confers hepatoprotection against ischaemia-reperfusion injury by suppressing TGFbeta1 via inhibition of CYR61/NF-kappaB in mouse hepatocytes. J. Cell Mol. Med.

[36] Shi Z (2021). Hypoxia-induced Nur77 activates PI3K/Akt signaling via suppression of Dicer/let-7i-5p to induce epithelial-to-mesenchymal transition. Theranostics.

[37] Wight L, Owen D, Goldbloom D, Knupp M (2017). Pure Ankle Dislocation: a systematic review of the literature and estimation of incidence. Injury.

[38] Jiang Y (2021). KDM6B-mediated histone demethylation of LDHA promotes lung metastasis of osteosarcoma. Theranostics.

[39] Mallaney C (2019). Kdm6b regulates context-dependent hematopoietic stem cell self-renewal and leukemogenesis. Leukemia.

[40] Li J (2021). KDM6B-dependent chromatin remodeling underpins effective virus-specific CD8(+) T cell differentiation. Cell Rep..

[41] Chang L (2021). H3K27 demethylase KDM6B aggravates ischemic brain injury through demethylation of IRF4 and Notch2-dependent SOX9 activation. Mol. Ther. Nucleic Acids.

[42] Maleszewska M, Vanchin B, Harmsen MC, Krenning G (2016). The decrease in histone methyltransferase EZH2 in response to fluid shear stress alters endothelial gene expression and promotes quiescence. Angiogenesis.

[43] Zhang Z (2022). Mechanical force-sensitive lncRNA SNHG8 inhibits osteogenic differentiation by regulating EZH2 in hPDLSCs. Cell Signal.

[44] Datta, H. K. et al. Mechanical-Stress-Related Epigenetic Regulation of ZIC1 Transcription Factor in the Etiology of Postmenopausal Osteoporosis. Int. J. Mol. Sci. **23**, 10.3390/ijms23062957 (2022). 10.3390/ijms23062957 PMC8955993 35328378

[45] Zheng T (2021). Long non-coding RNA HOTAIRincreased mechanical stimulation-induced apoptosis by regulating microRNA-221/BBC3 axis in C28/I2 cells. Bioengineered.

[46] Huang Z (2021). BRD4 inhibition alleviates mechanical stress-induced TMJ OA-like pathological changes and attenuates TREM1-mediated inflammatory response. Clin. Epigenet..

[47] Yasui T (2011). Epigenetic regulation of osteoclast differentiation: possible involvement of Jmjd3 in the histone demethylation of Nfatc1. J. Bone Min. Res..

[48] LaMere SA (2017). H3K27 Methylation Dynamics during CD4 T Cell Activation: Regulation of JAK/STAT and IL12RB2 Expression by JMJD3. J. Immunol..

[49] Li Q (2014). Critical role of histone demethylase Jmjd3 in the regulation of CD4+ T-cell differentiation. Nat. Commun..

[50] Huang M (2020). Jmjd3 regulates inflammasome activation and aggravates DSS-induced colitis in mice. FASEB J..

[51] Donas C (2016). The histone demethylase inhibitor GSK-J4 limits inflammation through the induction of a tolerogenic phenotype on DCs. J. Autoimmun..

[52] Hou KK, Pan H, Ratner L, Schlesinger PH, Wickline SA (2013). Mechanisms of nanoparticle-mediated siRNA transfection by melittin-derived peptides. ACS Nano.

[53] Zhou HF (2014). Peptide-siRNA nanocomplexes targeting NF-kappaB subunit p65 suppress nascent experimental arthritis. J. Clin. Investig..

[54] Yan H (2016). Suppression of NF-kappaB activity via nanoparticle-based siRNA delivery alters early cartilage responses to injury. Proc. Natl Acad. Sci. USA.

[55] Winoto A, Littman DR (2002). Nuclear hormone receptors in T lymphocytes. Cell.

[56] Lee JM, Lee KH, Weidner M, Osborne BA, Hayward SD (2002). Epstein-Barr virus EBNA2 blocks Nur77- mediated apoptosis. Proc. Natl Acad. Sci. USA.

[57] Zhou F (2014). Nuclear receptor NR4A1 promotes breast cancer invasion and metastasis by activating TGF-beta signalling. Nat. Commun..

[58] Maijenburg MW, van der Schoot CE, Voermans C (2012). Mesenchymal stromal cell migration: possibilities to improve cellular therapy. Stem Cells Dev..

[59] Landry ES, Rouillard C, Levesque D, Guertin PA (2006). Profile of immediate early gene expression in the lumbar spinal cord of low-thoracic paraplegic mice. Behav. Neurosci..

[60] Herring, J. A., Elison, W. S. & Tessem, J. S. Function of Nr4a Orphan Nuclear Receptors in Proliferation, Apoptosis and Fuel Utilization Across Tissues. *Cells* **8**, 10.3390/cells8111373 (2019). 10.3390/cells8111373 PMC6912296 31683815

[61] Shi X, Ye H, Yao X, Gao Y (2017). The involvement and possible mechanism of NR4A1 in chondrocyte apoptosis during osteoarthritis. Am. J. Transl. Res..

[62] Zheng Z (2020). NR4A1 promotes TNFalphainduced chondrocyte death and migration injury via activating the AMPK/Drp1/mitochondrial fission pathway. Int J. Mol. Med..

[63] Jin Y (2021). Aberrant Fluid Shear Stress Contributes to Articular Cartilage Pathogenesis via Epigenetic Regulation of ZBTB20 by H3K4me3. J. Inflamm. Res..

[64] Ruan MZ, Patel RM, Dawson BC, Jiang MM, Lee BH (2013). Pain, motor and gait assessment of murine osteoarthritis in a cruciate ligament transection model. Osteoarthr. Cartil..

[65] Pritzker KP (2006). Osteoarthritis cartilage histopathology: grading and staging. Osteoarthr. Cartil..

