# Supplementary material for: Histone demethylase JMJD3 downregulation protects against aberrant force-induced osteoarthritis through epigenetic control of NR4A1
Source: Int J Oral Sci. 2022 Jul 14;14:34. doi: 10.1038/s41368-022-00190-4 (PMC9279410; doi:10.1038/s41368-022-00190-4)
Supplement: Supplementary file 1 — supplemental materials [file 41368_2022_190_MOESM1_ESM.pdf]

Table S1. The primers used for real-time PCR analysis.

Genes	Primer sequences
Mouse SOX9	forward 5’-GAGGAAGTCGGTGAAGAACG-3’
	reverse 5’-CTGAGATTGCCCAGAGTGCT-3’
Mouse COLII	forward 5’-GTGGAGCAGCAAGAGCAAG-3’
	reverse 5’-CGGAGGAAAGTCATCTGGAC-3’
Mouse COX-2	forward 5’-GCAGGAAGTCTTTGGTCTGG-3’
	reverse 5’-AGTTGCTCATCACCCCACTC-3’
Mouse MMP13	forward 5’-CTTCTTCTTGTTGAGCTGGACTC-3’
	reverse 5’-CTGTGGAGGTCACTGTAGACT-3’
Mouse IL-1β	forward 5’- CCCAACTGGTACATCAGCACCTC-3’
	reverse 5’- GACACGGATTCCATGGTGAAGTC-3’
Mouse IL-6	forward 5’- ATAGTCCTTCCTACCCCAATTTCC-3’
	reverse 5’- GATGAATTGGATGGTCTTGGTCC-3’
Mouse TNF-α	forward 5’- CTGAACTTCGGGGTGATCGG-3’
	reverse 5’- GGCTTGTCACTCGAATTTTGAGA-3’
Mouse NR4A1	forward 5’- TTGAGTTCGGCAAGCCTACC-3’
	reverse 5’- GTGTACCCGTCCATGAAGGTG-3’
Mouse JMJD3	forward 5’- TGAAGAACGTCAAGTCCATTGTG-3’
	reverse 5’- TCCCGCTGTACCTGACAGT-3’
Mouse GAPDH	forward 5’-TTCCAGGAGCGAGACCCCACTA-3’
	reverse 5’-GGGCGGAGATGATGACCCTTTT-3’

Supplementary figures

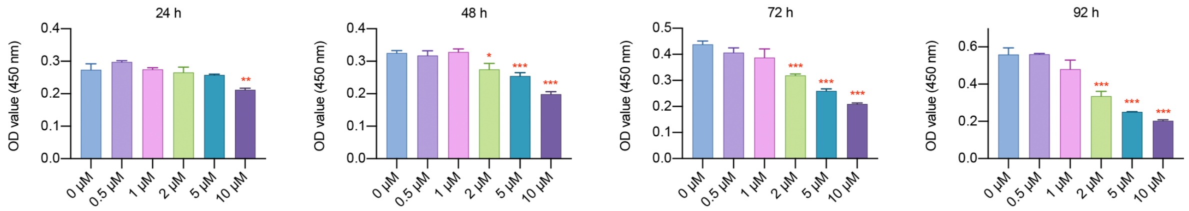

Fig. S1. CCK8 assay of primary chondrocytes treated with different concentrations of GSK-J4.

*p < 0.05, **p < 0.01, ***p < 0.001.

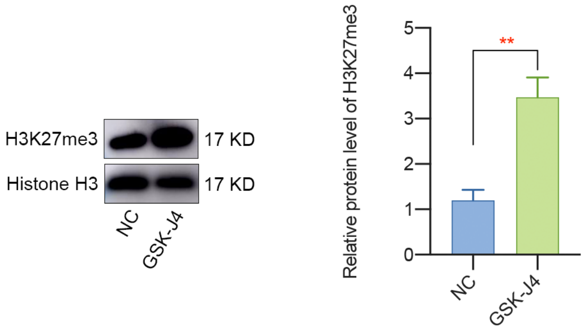

Fig. S2. Primary chondrocytes treated with 1μM GSK-J4 for 24 h led to an

upregulated expression level of H3K27me3. **p < 0.01.

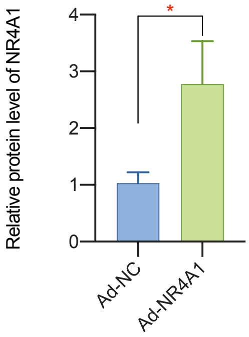

Fig. S3. Quantitative statistical analysis of western blot of Figure 5B. * p < 0.05.

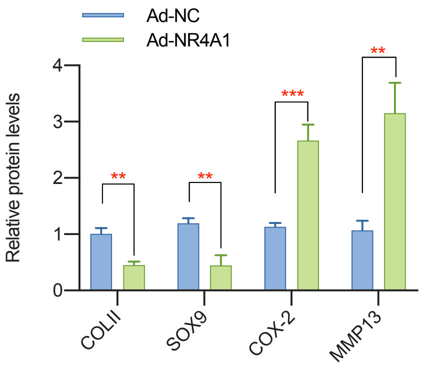

Fig. S4. Quantitative statistical analysis of western blot of Figure 5E. **p < 0.01; ***p < 0.001.

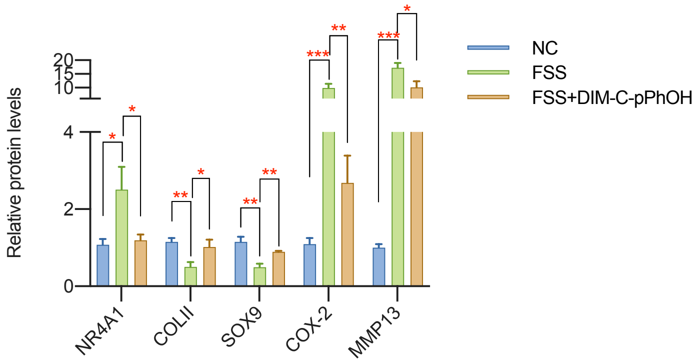

Fig. S5. Quantitative statistical analysis of western blot of Figure 5H.

* p < 0.05; **p < 0.01; ***p < 0.001.

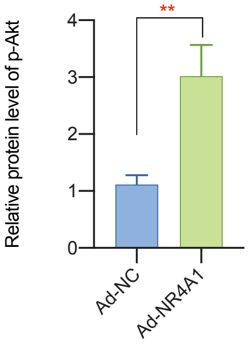

Fig. S6. Quantitative statistical analysis of western blot of Figure 6A. **p < 0.01.

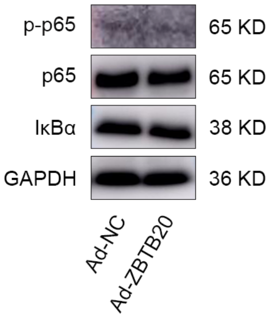

Fig. S7. NR4A1 overexpression had no mediatory functions on NF-κB pathway.

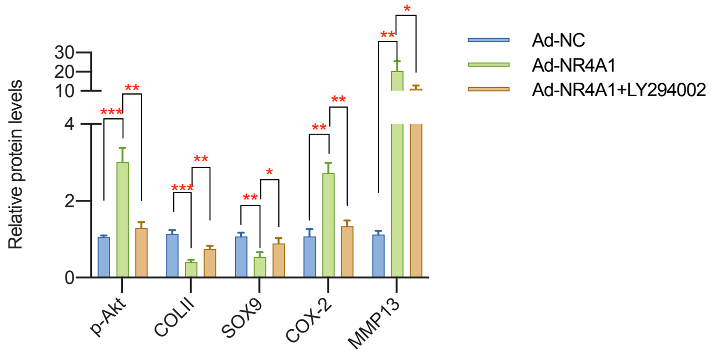

Fig. S8. Quantitative statistical analysis of western blot of Figure 6D.

* p < 0.05; **p < 0.01; ***p < 0.001.

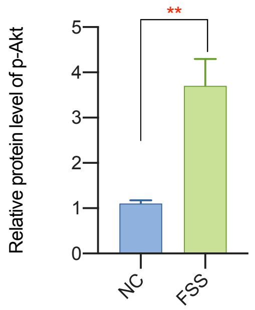

Fig. S9. Quantitative statistical analysis of western blot of Figure 6E. **p < 0.01

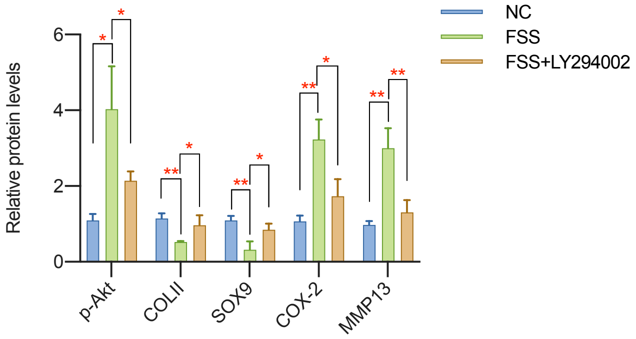

Fig. S10. Quantitative statistical analysis of western blot of Figure 6H. * p < 0.05; **p < 0.01.
